# A Phase 1b/2 trial of mapatumumab in patients with relapsed/refractory non-Hodgkin's lymphoma

**DOI:** 10.1038/sj.bjc.6605987

**Published:** 2010-11-16

**Authors:** A Younes, J M Vose, A D Zelenetz, M R Smith, H A Burris, S M Ansell, J Klein, W Halpern, R Miceli, E Kumm, N L Fox, M S Czuczman

**Affiliations:** 1 Lymphoma/Myeloma, MD Anderson Cancer Center, 1515 Holcombe Boulevard, Houston, TX 77030-4009, USA; 2 Hematology/Oncology, University of Nebraska Medical Center, Omaha, NE, USA; 3 Lymphoma Service, Memorial Sloan Kettering Cancer Center, New York City, NY, USA; 4 Medical Oncology, Fox Chase Cancer Center, Philadelphia, PA, USA; 5 Oncology, The Sarah Cannon Research Institute, Nashville, TN, USA; 6 Hematology, Mayo Clinic College of Medicine, Rochester, MN, USA; 7 Human Genome Sciences, Inc., Rockville, MD, USA; 8 Genentech, a Member of the Roche Group, South San Francisco, CA, USA; 9 Medicine, Roswell Park Cancer Institute, Buffalo, NY, USA

**Keywords:** TRAIL, apoptosis, Apo2L

## Abstract

**Background:**

We conducted a multicentre Phase 1b/2 trial to evaluate the safety and efficacy of mapatumumab, a fully human agonistic monoclonal antibody to the tumour necrosis factor-related apoptosis-inducing ligand receptor 1 (TRAIL-R1) in patients with relapsed non-Hodgkin's lymphoma (NHL).

**Methods:**

Forty patients with relapsed or refractory NHL were treated with either 3 or 10 mg kg^−1^ mapatumumab every 21 days. In the absence of disease progression or prohibitive toxicity, patients received a maximum of six doses.

**Results:**

Mapatumumab was well tolerated, with no patients experiencing drug-related hepatic or other dose-limiting toxicity. Three patients with follicular lymphoma (FL) experienced clinical responses, including two with a complete response and one with a partial response. Immunohistochemistry staining of the TRAIL-R1 suggested that strong staining in tumour specimens did not appear to be a requirement for mapatumumab activity in FL.

**Conclusions:**

Mapatumumab is safe and has promising clinical activity in patients with FL.

Mapatumumab (HGS-ETR1, TRM-1) is a fully human IgG_1_ monoclonal antibody specific and agonistic to the tumour necrosis factor-related apoptosis-inducing ligand receptor 1 (TRAIL-R1, DR4). The TRAIL-R1 is expressed more frequently on the surface of tumour cells than on the surface of normal cells (Zang *et al*, 2001; Koornstra *et al*, 2003). Mapatumumab, like the native ligand TRAIL, mediates apoptosis by binding to TRAIL-R1, leading to activation of the caspase cascade, cleavage of key intracellular signalling components and DNA and subsequent cell death (Pukac *et al*, 2005). Mapatumumab induces apoptosis in a wide range of haematologic and solid tumour cell lines and xenograft models (Snell *et al*, 1997; Georgakis *et al*, 2005; Pukac *et al*, 2005; Menoret *et al*, 2006). In addition to a direct induction of apoptosis, mapatumumab may also induce antibody-dependent cellular cytotoxicity. Furthermore, *in vivo* experiments using disseminated B cell lymphoma models showed that mapatumumab in combination with rituximab increased animal survival compared with control, rituximab alone or mapatumumab alone (Maddipatla *et al*, 2007).

Mapatumumab was evaluated first as a single agent and subsequently in combination with chemotherapeutic agents in a series of Phases 1, 1b and 2 clinical trials (Tolcher *et al*, 2007; Greco *et al*, 2008; Hotte *et al*, 2008; Mom *et al*, 2009; Trarbach *et al*, 2010). In general, it is well tolerated both as a single agent and in combination with chemotherapy. No disease response was observed in the other single-agent trials. This clinical trial was designed to evaluate single-agent mapatumumab in non-Hodgkin's lymphoma (NHL). The doses and design were based on data available from the initial and on-going Phase 1 trials of mapatumumab (Tolcher *et al*, 2007; Hotte *et al*, 2008).

## Materials and methods

### Patients

Eligibility criteria included histologically confirmed NHL with measurable disease ⩾1.5 cm in the longest transverse diameter by computed tomography scan. Patients were previously treated with at least one therapeutic regimen and had relapsed or progressed, or failed to achieve an objective response after their last therapeutic regimen. Adequate haematologic, bone marrow, hepatic and renal functions, and an Eastern Cooperative Oncology Group performance status 0–2 were required. Patients were excluded if they had been treated with a monoclonal antibody or radioimmunotherapy within 8 weeks or had persistent clinical evidence of toxicity, were eligible for stem cell transplantation (SCT), had undergone autologous SCT within 16 weeks or had ever undergone allogeneic SCT. The IRB-approved, written informed consent was obtained from all patients.

### Study design

This multicentre, open-label study was designed to evaluate the safety and efficacy of mapatumumab in patients with relapsed or refractory NHL. At the time this study was initiated, safety data were available from on-going Phase 1 trials for doses up to 3 mg kg^−1^ every 28 days. This trial was designed to allow for treatment of a small number of NHL patients with 3 mg kg^−1^ every 21 days before treating a larger number of patients with 10 mg kg^−1^ every 21 days. Specifically, safety data from eight patients treated with 3 mg kg^−1^ mapatumumab were reviewed before enrolment of the 10 mg kg^−1^ cohort. Mapatumumab was administered intravenously (i.v.) over 2 h in a total volume of 250 ml normal saline. No dose reduction was allowed. Premedication with diphenhydramine and acetaminophen was allowed and usually given. Patients were treated on day 1 of each 21(±2)-day cycle for up to six cycles in the absence of disease progression or dose-limiting toxicity. Patients who demonstrated a tumour response after six cycles of treatment could receive permission to continue treatment. Disease assessment was performed at baseline, and after cycles three and six, and then every 3-month intervals until disease progression. Disease response was evaluated using response criteria according to the International Working Group Recommendations for NHL (Cheson *et al*, 1999). Adverse events (AEs) and clinical laboratory test results were graded based on the NCI-CTCAE (Version 3.0).

Plasma mapatumumab concentrations were measured before dosing, immediately post-dosing and 4–6 h post-dosing for the first mapatumumab administration. Additional sampling was performed before dosing, immediately post-dosing and 24 h post-dosing after the second administration, and before dosing and day 17 (day of disease assessment) for the third and sixth administration. Anti-mapatumumab antibodies were analysed using an enzyme-linked immunosorbent assay. Serum samples were obtained at baseline, prior to dosing in each treatment cycle, at cycle 1, day 17 and 28–30 days after the last dose of mapatumumab. Formalin-fixed, paraffin-embedded tissue blocks or 4–6 mm tissue sections from biopsies collected at baseline, post-dose or from archival specimens were requested from all patients and collected as available. The immunohistochemistry (IHC) staining method utilised standardised reagents according to a protocol supplied by DAKO (Carpinteria, CA, USA) for this prototype pharmDx test intended for investigative use (Humphreys and Halpern, 2008).

### Statistical considerations

Progression-free survival was calculated from baseline to disease progression (or death) using the Kaplan–Meier method. To assess potential relationships between AEs and plasma mapatumumab concentrations, dose normalised plasma mapatumumab concentrations at selected time points were compared among patients who experienced serious adverse event (SAEs) and those who did not, only if *n*⩾3 for each subgroup, using two-sided unpaired *t*-tests at a significance level of *a*=0.05.

## Results

**Figure 1:**
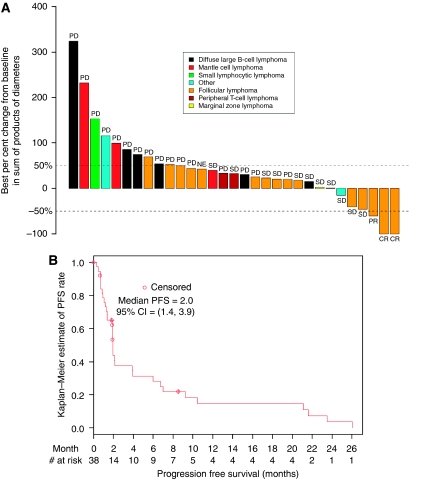
(**A**) Best per cent change from baseline in sum of products of diameters (SPD). Nine patients lacking a complete post-baseline disease assessment are excluded from the figure. (**B**) Progression-free survival, all patients (*n*=40).

Among the 40 patients treated in this study, 8 received 3 mg kg^−1^ mapatumumab and 32 received 10 mg kg^−1^ mapatumumab. Patient demographics and baseline disease characteristics are summarised in Table 1. The majority of patients (26 out of 40, 68%) completed at least 3 cycles and 12 patients received the full 6 cycles. Two patients receiving 10 mg kg^−1^ demonstrated a tumour response after six cycles and received permission to continue mapatumumab until disease progression. One patient received 12 cycles, discontinued the study for an unrelated medical condition and, at the time of discontinuation, had an increase in tumour size not yet reaching disease progression. The other patient received 27 cycles before disease progression. Three patients with follicular lymphoma (FL) (Grade 1 or 2) achieved major responses: one complete response (CR) in the 3 mg kg^−1^ cohort, and one partial response (PR) and one CR in the 10 mg kg^−1^ cohort. The median time to response (CR or PR) was 3.9 months (range: 1.8–4.1 months). The patient in the 3-mg kg^−1^ cohort achieved a PR after completing six cycles of treatment; her tumour continued to shrink, and she achieved a CR at 11.4 months. The mechanism of this continued response is unknown, but based on the plasma half-life of mapatumumab, the subject would have been exposed to mapatumumab for several months after discontinuing drug. This patient had disease progression at 26 months. The two patients in the 10 mg kg^−1^ cohort who achieved disease response after six cycles received IRB permission to continue on mapatumumab. The patient with the PR received a total of 12 cycles and was taken off study to allow for treatment of bilateral urethral stones. This patient had progressing disease at the time of discontinuation at 8.5 months with a 45.9% increase in tumour. The second patient received 27 cycles and converted from a PR to a CR at 9.3 months. The patient exhibited progressive disease after 21 months. A total of six patients experienced tumour shrinkage following mapatumumab therapy (Figure 1A). The median PFS for all patients in the study was 2 months (Figure 1B). For the subset of 17 FL patients, the median PFS was 6 months (95% CI: 2.1–21 months). Two patients with FL demonstrated durable stable disease after six cycles with a PFS of 24 and 21 months, respectively.

The most commonly reported AEs, regardless of relationship to study drug, are shown in Table 2. Most of these events were Grade 1 or 2 in severity. The most common AEs considered at least possibly related to mapatumumab included nausea, fatigue, diarrhoea, anorexia and pyrexia. These events were Grade 1 or 2 with the exception of a single event of Grade 3 pyrexia. Eleven patients experienced 29 SAEs. Three SAEs reported in the 10 mg kg^−1^ cohort were considered either possibly, probably or definitely related to treatment with mapatumumab. One patient experienced peripheral, facial and angioneurotic oedema, and discontinued treatment. The other two SAEs, pyrexia and herpes zoster occurred in a single patient each. Laboratory data showed no evidence of significant hepatotoxicity or renal toxicity. One patient developed Grade 3 GGT levels. One developed Grade 2 hyperbilirubinemia. Two patients developed hyperamylasemia (Grade 3 or 4). No patient developed anti-mapatumumab antibodies.

**Figure 2:**
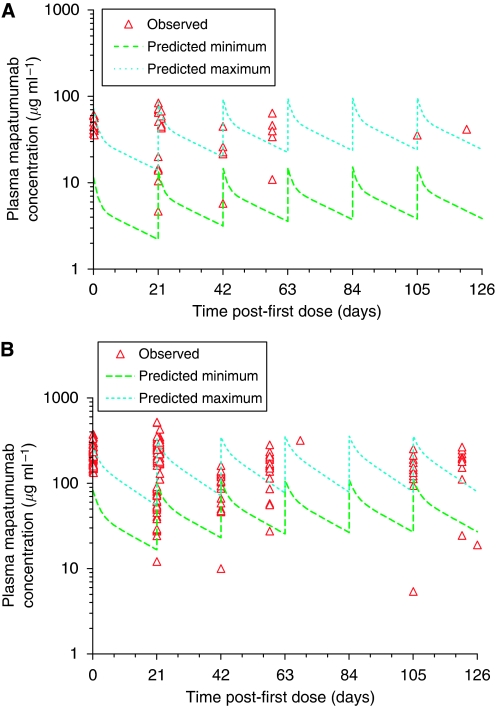
Plasma mapatumumab concentrations observed for individual patients following 3 or 10 mg kg^−1^ mapatumumab i.v. infusion doses given 21 days apart, with the expected minimum to maximum concentration range based on Phase 1 study results. (**A**) 3 mg kg^−1^ mapatumumab and (**B**) 10 mg kg^−1^ mapatumumab.

The mean serum mapatumumab concentration–time profiles are illustrated in Figure 2. The observed plasma mapatumumab concentrations tended to be higher than those predicted from the Phase 1 trials of mapatumumab in solid tumours and is due to a slower clearance of mapatumumab in these study patients (Tolcher *et al*, 2007; Hotte *et al*, 2008). The terminal elimination half-life (*t*_1/2,*z*_) was ∼26 days in NHL patients compared with 12–24 days seen in patients with solid tumours (Tolcher *et al*, 2007; Hotte *et al*, 2008).

Twenty-four patients provided a total of 29 evaluable tissue specimens: 18 archival specimens, 7 specimens before treatment and 4 post-treatment. Of these, TRAIL-R1 was detected in 16 specimens from 14 patients, with at least 10% of tumour cells staining. Tumours with an aggressive phenotype demonstrated the strongest TRAIL-R1 staining. The TRAIL-R1-specific staining was not detected or was weak in the archival or baseline specimens from two of the three patients with FL who responded to mapatumumab; biopsy material from the third patient was not evaluable.

## Discussion

This is the first trial to demonstrate disease response from single-agent mapatumumab in lymphoma. The results introduce three important questions: (1) what dose(s) of mapatumumab should be evaluated in future studies of mapatumumab in NHL; (2) whether staining for TRAIL-R1 by IHC can help identify patients most likely to benefit from mapatumumab and (3) whether mapatumumab should be further evaluated as a single agent for NHL or in combination with other therapeutics that target relapsed or refractory NHL.

At the time this study was initiated, safety data were available from on-going Phase 1 trials in advanced solid tumour patients for doses up to 3 mg kg^−1^ every 28 days. Preclinical models showed mapatumumab to be active in doses that correspond to 3 and 10 mg kg^−1^. Based on the availability of data from Phase 1 solid tumour trials and the preclinical models, this trial was designed to allow for treatment of a small number of NHL patients with 3 mg kg^−1^ before treating a larger number of patients with 10 mg kg^−1^. In subsequent clinical trials, doses as high as 30 mg kg^−1^ have been administered to solid tumour patients in combination with chemotherapy. Whether higher doses of mapatumumab would have led to more patients achieving a CR or PR is unknown, but safety data from other trials support further evaluation of higher doses. As TRAIL-R1 is expressed more widely on the surface of tumour cells than normal cells, staining by IHC for TRAIL-R1 in tissue specimens of the lymphoma was of special interest. It is noteworthy that for two patients who experienced a PR or CR, TRAIL-R1 staining was not detected or was weak. Thus, strong staining in tumour specimens does not appear to be a prerequisite for mapatumumab in FL.

Although three patients with FL experienced a CR or PR in response to single-agent mapatumumab, the first demonstration of disease response from single-agent mapatumumab in lymphoma, the biology of the TRAIL receptors, preclinical studies of mapatumumab and accruing clinical data emphasise that mapatumumab is likely to be most efficacious when administered in combination with other agents, notably agents that affect the downstream ‘checks and balances’ of the apoptosis pathways (Jin *et al*, 2007; Maddipatla *et al*, 2007; Smith *et al*, 2007). It is noteworthy that dulanermin, Apo2L/TRAIL, which activates the extrinsic pathway through both TRAIL-R1 and TRAIL-R2, did not improve overall response when combined with rituximab in patients with relapsed FL (Belada *et al*, 2010). Accordingly, on-going randomised Phase 2 trials of mapatumumab in combination with bortezomib and sorafenib will be highly informative with regard to the selection of agents to combine with mapatumumab and the design of additional clinical trials of mapatumumab including studies in relapsed or refractory FL.

## Figures and Tables

**Table 1:** Patient demographic and baseline disease characteristics

	**3 mg kg^–1^ (*n*=8)**	**10 mg kg^–1^ (*n*=32)**	**Total (*n*=40)**
*Sex*			
Male	4 (50%)	20 (63%)	24 (60%)
Female	4 (50%)	12 (38%)	16 (40%)
			
*Race*			
White	7 (88%)	31 (97%)	38 (95%)
Black or African American	1 (13%)	1 (3%)	2 (5%)
			
*Age (years)*			
Median (range)	63 (51–74)	57 (32–81)	60 (32–81)
			
*Time from first diagnosis (months)*			
Median (range)	36 (5–88)	40.5 (9–322)	40 (5–322)
			
*Number of prior systemic regimens*			
Median (range)	3.5 (1–12)	3.0 (1–9)	3.0 (1–12)
			
Rituximab – combination or single agent (B-cell lymphoma patients)	8/8 (100%)	26/28 (93%)	34/36 (94%)
			
*ECOG performance status*			
0	3 (38%)	19 (60%)	22 (55%)
1	5 (63%)	11 (34%)	16 (40%)
2		2 (6%)	2 (5%)
			
*Histological classification at diagnosis*			
Small lymphocytic		1 (3%)	1 (3%)
Marginal zone		1 (3%)	1 (3%)
*Follicular*	2 (25%)	15 (47%)	17 (43%)
Grade 1		7 (22%)	7 (18%)
Grade 2	2 (25%)	6 (19%)	8 (20%)
Grade 3		1 (3%)	1 (3%)
Unclassified		1 (3%)	1 (3%)
Mantle cell	3 (38%)	3 (9%)	6 (15%)
Diffuse large B cell	2 (25%)	7 (22%)	9 (23%)
Anaplastic large cell		1 (3%)	1 (3%)
Peripheral T cell		3 (9%)	3 (8%)
B cell unclassified	1 (13%)	1 (3%)	2 (5%)

Abbreviation: ECOG=Eastern Cooperative Oncology Group.

**Table 2:** Number of patients with treatment–emergent AEs by severity regardless of relationship to study drug (for ⩾10% of subjects, *n*=40)

**Preferred term**	**Grade 1**	**Grade 2**	**Grade 3**	**Grade 4**
Fatigue	12 (30.0%)	7 (17.5%)	—	—
Nausea	9 (22.5%)	4 (10.0%)	1 (2.5%)	—
Diarrhoea	12 (30.0%)	—	—	—
Pyrexia	8 (20.0%)	2 (5.0%)	1 (2.5%)	—
Oedema peripheral	7 (17.5%)	3 (7.5%)	—	—
Anorexia	6 (15.0%)	2 (5.0%)	—	—
Constipation	5 (12.5%)	3 (7.5%)	—	—
Upper respiratory tract infection	4 (10.0%)	3 (7.5%)	1 (2.5%)	—
Vomiting	4 (10.0%)	3 (7.5%)	1 (2.5%)	—
Back pain	1 (2.5%)	4 (10.0%)	2 (5.0%)	—
Cough	6 (15.0%)	1 (2.5%)	—	—
Abdominal pain	2 (5.0%)	1 (2.5%)	3 (7.5%)	—
Dizziness	6 (15.0%)	—	—	—
Dyspnoea	4 (10.0%)	1 (2.5%)	—	1 (2.5%)
Chills	5 (12.5%)	—	—	—
Dyspepsia	4 (10.0%)	1 (2.5%)	—	—
Headache	3 (7.5%)	1 (2.5%)	1 (2.5%)	—
Lymph node pain	4 (10.0%)	1 (2.5%)	—	—
Pain in extremity	2 (5.0%)	2 (5.0%)	1 (2.5%)	—
Pruritus	3 (7.5%)	2 (5.0%)	—	—
Weight decreased	4 (10.0%)	1 (2.5%)	—	—
Anaemia	—	2 (5.0%)	2 (5.0%)	—
Asthenia	4 (10.0%)	—	—	—
Hyperglycaemia	1 (2.5%)	3 (7.5%)	—	—
Muscle spasms	3 (7.5%)	1 (2.5%)	—	—
Insomnia	4 (10%)	—	—	—

Abbreviation: AEs=adverse events.
